# Fusion of GFP to the M.EcoKI DNA methyltransferase produces a new probe of Type I DNA restriction and modification enzymes

**DOI:** 10.1016/j.bbrc.2010.06.069

**Published:** 2010-07-23

**Authors:** Kai Chen, Gareth A. Roberts, Augoustinos S. Stephanou, Laurie P. Cooper, John H. White, David T.F. Dryden

**Affiliations:** School of Chemistry, University of Edinburgh, The King’s Buildings, Edinburgh, EH9 3JJ, UK

**Keywords:** DNA restriction/modification, DNA methyltransferase, Forster resonance energy transfer, Time-resolved fluorescence anisotropy, Time-resolved fluorescence, Green fluorescent protein

## Abstract

We describe the fusion of enhanced green fluorescent protein to the C-terminus of the HsdS DNA sequence-specificity subunit of the Type I DNA modification methyltransferase M.EcoKI. The fusion expresses well *in vivo* and assembles with the two HsdM modification subunits. The fusion protein functions as a sequence-specific DNA methyltransferase protecting DNA against digestion by the EcoKI restriction endonuclease. The purified enzyme shows Förster resonance energy transfer to fluorescently-labelled DNA duplexes containing the target sequence and to fluorescently-labelled ocr protein, a DNA mimic that binds to the M.EcoKI enzyme. Distances determined from the energy transfer experiments corroborate the structural model of M.EcoKI.

## Research highlights

► Successful fusion of GFP to M.EcoKI DNA methyltransferase. ► GFP located at C-terminal of sequence specificity subunit does not later enzyme activity. ► FRET confirms structural model of M.EcoKI bound to DNA.

## 1 Introduction

Since their introduction into genetic engineering, the green fluorescent protein (GFP) and its many spectral variants have proved to be extraordinarily useful probes of protein structure and function both *in vitro* and *in vivo* [1]. In particular, Förster resonance energy transfer (FRET) to measure distances between two fluorophores, a donor and an acceptor, has been the subject of many uses of GFP despite its complex photophysics and its relatively large size compared to more traditional small molecule fluorophores such as fluorescein [2].

Sequence-specific DNA-binding enzymes such as methyltransferases (MTases) and endonucleases comprising bacterial restriction–modification (R/M) systems would seem to present excellent targets for analysis via fusion to GFP given that many of them introduce complex rearrangements of DNA structure including for example DNA looping to bring distant sites on a single DNA molecule into close proximity. However, as yet few investigations of R/M systems have utilised these versatile fluorescent probes [3].

Bacterial host restriction endonucleases (REase) attack invading foreign DNA lacking the imprinted modification pattern characteristic of the host DNA [4]. R/M systems typically comprise a REase that recognises a specific nucleotide sequence prior to cleavage, and a cognate DNA MTase able, by methylating adenine or cytosine within the same sequence, to confer protection from the REase. The REase cuts unmethylated DNA but not hemimethylated DNA, the substrate for the MTase. R/M systems are classified according to their subunit composition, recognition site, cofactor requirement and DNA cleavage position. The R/M systems display an extraordinary diversity in structure and activity leading to four distinct groupings [5]. The most common R/M systems are the Type II R/M systems, which primarily consist of separate MTase and REase enzymes that recognise 4–8 base pair (bp) palindromic sequences.

In contrast, Type I R/M enzymes [4] such as EcoKI are complex hetero-oligomers of two REase (HsdR) subunits, two MTase (HsdM) subunits and one DNA sequence-specificity (HsdS) subunit. Depending on the methylation status of the DNA substrate, this complex functions as either a REase or an MTase. These enzymes recognise an asymmetric, bipartite sequence (13–15 bp) and require ATP to affect cleavage at a distant site. Over 600 confirmed and putative Type I R/M systems are known and they appear to be as widely spread in bacteria as the Type II R/M systems [6]. The complex of 2 HsdM and 1 HsdS, M_2_S_1_, forms an active MTase, M.EcoKI, and is the core part of the Type I R/M enzyme. The M.EcoKI MTase recognises the sequence AACNNNNNNGTGC and the methylation status of the adenines at the underline locations. A detailed structural model of M.EcoKI in complex with DNA has recently been proposed based upon electron microscopy of the complex and crystallographic structures of the individual subunits [7].

The genes for R/M systems are found in virtually every sequenced bacterial and archaeal genome and many genomes contain multiple R/M systems [6] often with the capability to switch between different systems and DNA specificities depending upon conditions [8,9]. R/M systems are also extensively represented within clinical strain collections such as the *Escherichia coli* ECOR collection [10]. Given that resident R/M systems limit phage propagation in a bacterial population by factors reaching 10^8^ (for EcoKI), there is a huge evolutionary pressure on mobile genetic elements such as phage and conjugative plasmids and transposons to evolve ‘anti-restriction’ counter measures including, for example, the acquisition of proteins which inhibit DNA binding by the R/M enzymes [4]. These inhibitors are structural and electrostatic mimics of double stranded DNA with the gene 0.3 protein, ocr, from phage T7 and the ArdA protein from conjugative Tn*916* mimicking 24 base pairs and 42 base pairs, respectively [11,12]. Their tight binding to M.EcoKI physically fills the DNA binding groove on the enzyme resulting in the inactivation of the R/M system [13–17].

In this paper we demonstrate the preparation of an active M.EcoKI fused to GFP and measure via FRET the distance from the GFP to a HEX label on a duplex bound to the MTase and to a fluorescently-labelled ocr protein bound to the MTase. These distances are then compared to predictions from the structural model [7].

## 2 Materials and methods

### 2.1 Plasmid pJFMSEGFP for production of GFP-MTase

The expression construct is derived from pJFMS [18] and pEGFP-N1 (Clonetech) as detailed in supplementary information. This plasmid was named pJFMSEGFP and we call the protein GFP-MTase.

### 2.2 *In vivo* activity

pJFMSEGFP and control plasmid pBIO2 were introduced into the r^−^m^−^ mutant, *E. coli* NM1261(DE3). This strain contains a mutation in *hsdS*. pBIO2 is a non-functional derivative of pJFMS lacking the entire *hsdM* and half of *hsdS*. In NM1261(DE3), function of the MTase was dependent upon the plasmid-encoded HsdS forming a complex with HsdM encoded on the chromosome and the plasmid. Bacteriophage lambda virulent containing unmodified EcoKI sites:*λ*_v.o_) was plated on NM1261(DE3) pJFMSEGFP and plaques were picked for assay against the EcoKI tester stains *E. coli* NM1049(DE3) (r^+^m^+^) and NM1261(DE3). Serial dilutions of plaques resuspended in phage buffer were spotted in 10 μl aliquots on the tester strains plated on BBL top agar supplemented with carbenicillin, 100 μg/ml. Titres were scored after overnight incubation at 37 °C [19]. Note that heterologous gene expression was not induced by addition of IPTG in these experiments but instead relied upon leaky expression from the promoter.

### 2.3 Purification of GFP-MTase

GFP-MTase was purified to homogeneity after overexpression in *E. coli* BL21(DE3) cells using His-tag affinity, gel filtration and anion exchange chromatography as detailed in supplementary information. The protein occurred in both M_1_S_1_ and M_2_S_1_ forms as found for the native protein [18] with the M_2_S_1_ form being used in further experiments. All subsequent measurements were performed at 20 or 25 °C in 20 mM Tris–HCl pH 8.0, 6 mM MgCl_2_, 7 mM 2-mercaptoethanol supplemented with NaCl when stated.

### 2.4 DNA binding activity *in vitro*

DNA binding was measured using FRET and employed 21 base pair duplexes labelled at their 5′ ends with hexachlorofluorescein (HEX). The interaction of these duplexes with M.EcoKI has been previously analysed using fluorescence anisotropy [15]. Two duplexes were used: 21TH21B has the top “21TH” strand sequence 5′-HEX-GCC TAA CCA CGT GGT GCG TAC-3′ with the complementary unlabelled bottom strand (“21B”) and 21T21BH has the same sequence but the HEX label is on the 5′ end of the bottom “21BH” strand.

A range of solutions containing GFP-MTase from 0 to 200 nM and NaCl concentrations of 0, 25, 50 and 100 mM, were prepared. In addition, solutions containing different proportions of GFP-MTase and 21TH21B, where the sum of the concentration of the two components was 200 nM, were prepared. The emission spectrum of each solution was then recorded and the intensity of the emission peak plotted against the mole fraction of GFP-MTase after subtracting the intensity of the GFP-MTase alone. The binding affinity was determined using the continuous variation method [20].

### 2.5 Preparation of ocr mutant proteins and their interaction with GFP-MTase

Site directed mutagenesis and protein purification was performed as described previously [17] to create the single substitutions, E20C, S68C and E117C in the ocr protein. 1 ml samples of 10 μM of each mutant ocr protein (assuming an ocr dimer) were incubated overnight at 4 °C in the dark with a 20-fold molar excess of Dylight549 Maleimide (Molecular Probes) in 100 mM sodium phosphate buffer, 150 mM NaCl, 1 mM EDTA, pH 7.2. Unreacted probe was removed by extensive dialysis. The concentration of Dylight549 bound to the ocr dimer was calculated from absorption using a molar extinction coefficient of 150 000 M^−1^ cm^−1^ at 562 nm. The concentrations of all ocr mutant proteins were calculated using a molar extinction coefficient of 31,860 M^−1^ cm^−1^ at 280 nm for the ocr dimer [13]. The concentrations of the labelled proteins were calculated from the absorption spectra at 280 nm after subtracting the Dylight549 absorbance at this wavelength (12,150 M^−1^ cm^−1^). A comparison of the concentration of Dylight549 with the concentration of ocr then allowed the degree of labelling to be calculated. Labeling levels of 81.5%, 77.0% and 86.3% were achieved for E20C, S68C and E117C mutant ocr proteins, respectively.

Binding of the labelled mutant ocr proteins to the GFP-MTase was assessed using size exclusion chromatography as previously described [14].

### 2.6 Fluorescence measurements

Steady state fluorescence intensity measurements were performed on an Edinburgh Instruments FS900 spectrofluorometer (Edinburgh Instruments) with a 5 nm bandwidth. The cuvette path lengths were 3 mm.

Time correlated single photon counting was performed with a home built time-resolved fluorimeter equipped with an Edinburgh Instruments TCC900 single photon counting card, 465 nm or 500 nm pulsed LED driven by a PDL 800-B pulsed diode laser driver (PicoQuant Gmbh) and a PMH-100-3 single photon counting photomultiplier tube (Becker & Hickl Gmbh). A 405 nm pulsed laser (Edinburgh Instruments) was also sometimes used. Emission wavelengths were selected with a monochromator. Polarisation was applied using quartz Glan–Thompson polarisers. Excitation pathlengths were 10 mm and the emission bandpass was 20 nm. Fluorescence decays were fitted using a multiexponential decay equation with the minimum number of decay components required to obtain a *χ*^2^ value close to 1. Anisotropy decays were fitted to Eq. (1).

(1) $$r(t)=r_{\infty }+r_{o}\exp(-t/\phi )$$

where *r*(*t*) is the anisotropy value at time *t*, *r*_o_ is the initial anisotropy, $r_{\infty }$ is the anisotropy value at infinite time and *ϕ* is the rotational correlation time.

### 2.7 FRET calculations

The Förster distance for 50% transfer efficiency (*R*_0_) for GFP to HEX or Dylight549 was calculated on the basis of Eq. (2). [21]

(2) $$R_{0}^{6}=8.78*10^{-5}\kappa^{2}\Phi {\mathrm{Jn}}^{-4}$$

where *n* is the refractive index of the medium (*n* = 1.33), the orientation factor (*κ*^2^) was considered to be two-thirds on the assumption that the donor and acceptor can adopt random conformations, the quantum yield of GFP was *Φ* = 0.8. The spectral overlap integral, *J*, between the donor emission spectrum and the acceptor absorbance spectrum was determined by using Eq. (3),

(3) $$J(\lambda )=\int F_{\text{d}}(\lambda )\epsilon_{\text{a}}(\lambda )\lambda 4\text{d}\lambda /\int F_{\text{d}}(\lambda )\text{d}\lambda$$

where *F*_d_(*λ*) and *ε*_a_(*λ*) represent the fluorescence intensity of the donor and the molar extinction coefficient of the acceptor, respectively, at wavelength *λ*.

The efficiency of the energy transfer was calculated based on the decrease in the donor (GFP) fluorescence intensity, Eq. (4).

(4) $$E=1-(F_{\text{a}}/F_{\text{d}})$$

where *F*_a_ and *F*_d_ represent the donor fluorescence intensity measured in the absence and presence of acceptor, respectively.

The efficiency of the energy transfer was also calculated from the decrease in the fluorescence lifetime of the donor (GFP) fluorescence, Eq. (5).

(5) $$E=1-(\tau_{\text{a}}/\tau_{\text{d}})$$

where *τ*_a_ and *τ*_d_ are, respectively, the fluorescence lifetime in the absence and presence of acceptor.

## 3 Results

### 3.1 Protein overexpression

The structural model [9] predicts that the C-terminus of HsdS should be exposed to solvent and would therefore present a suitable site for fusion to the N-terminus of GFP. This fusion gene construct (pJFMSEGFP) was engineered and produced large amounts of GFP-MTase, which could be purified to homogeneity (see supplementary Figure S1).

### 3.2 *In vivo* activity

We tested whether the fusion had any effect on the activity of M.EcoKI *in vivo* using phage lambda. Expression of the GFP-MTase was sufficient to modify the five EcoKI target sites on *λ*_v.o_ as shown by the survival of these phage, when passaged through *E. coli* NM1261(DE3) pJFMSEGFP, on an EcoKI restriction proficient strain, NM1149(DE3), or a restriction deficient strain, NM1261(DE3), Table 1. The titre of phage isolated from NM1261(DE3) pJFMSEGFP, was the same on both the restricting and non-restricting strain. Thus the fusion does not interfere with the operation of M.EcoKI and the enzyme is still a functional sequence-specific MTase.

### 3.3 Absorption and fluorescence spectra

**Fig. 1:**
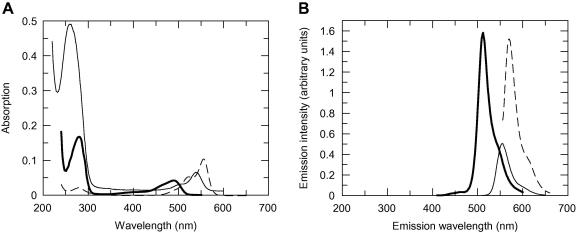
Spectrophotometric analyses. (A) Absorption spectra of 1 μM GFP-MTase (bold solid line), 1 μM 21TH21B DNA (thin solid line) and 5 μM Dylight549-labeled ocr E20C mutant protein (dashed line). Other labeled proteins had similar spectra. (B) Emission spectra of 1 μM GFP-MTase (bold solid line, excitation at 395 nm), 400 nM 21TH21B DNA (thin solid line, excitation at 530 nm) and 1 μM Dylight549-labeled ocr E20C mutant protein (dashed line, excitation at 550 nm). Other labeled proteins had similar spectra.

The purified GFP-MTase showed the absorption and fluorescence emission properties expected, Fig. 1A, B. The overlap of the emission of the GFP with the HEX and Dylight549 labels allowed *R*_o_ distances of 6.14 nm and 6.53 nm, respectively, to be calculated.

### 3.4 Fluorescence and anisotropy decay of the fluorescent labels

To use energy transfer quantitatively, one ideally should determine whether the donor and acceptor chromophores are free to rotate or are sterically hindered on the nanosecond timescale as this indicates that the *κ*^2^ orientation parameter can be reasonably set at 2/3 as assumed in Eq. (2). The time-resolved data, supplementary Table S1, indicated that the GFP was rotating on the nanosecond timescale despite its attachment to the MTase but that the degree of rotational freedom on GFP when fused to the MTase was slightly less than that of the free GFP as indicated by the higher value of the anisotropy at infinite time. The rotational correlation time of the HEX label on the DNA duplex was unaffected by GFP-MTase binding as was the degree of rotation of the Dylight549 label when attached to the E20C and E117C mutant ocr proteins, supplementary Table S1 and Figure S3. The label attached to the S68C mutant protein showed an unusual anisotropy decay shape in the absence of GFP-MTase and a long anisotropy decay time in the presence of the GFP-MTase, supplementary Figure S3. These data indicate that the label attached to the S68C position is not free to rotate and hence that the *κ*^2^ orientation parameter is not 2/3 in the FRET experiments.

### 3.5 DNA binding by GFP-MTase

We additionally checked that the assumption of 1:1 binding to DNA and ocr was correct for the GFP-MTase as previously established for the normal MTase. The interaction between GFP-MTase and both ligands was found to be the same as for the normal MTase [14,15], supplementary information and supplementary Figure S2. The use of concentrations more than 10-fold greater than the dissociation constants for DNA and ocr ensured that there was little unbound donor or acceptor to complicate FRET analysis.

### 3.6 Steady state fluorescence analysis of FRET between GFP-MTase, DNA and labelled ocr

**Fig. 2:**
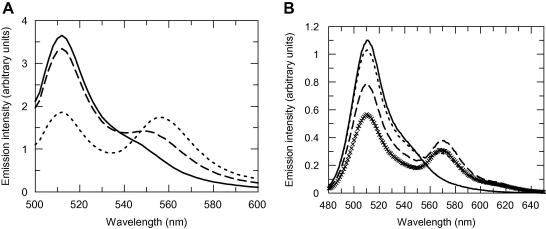
Fluorescence energy transfer. (A) Fluorescence emission scans of 200 nM GFP-MTase showing the effects of FRET to 200 nM HEX-labelled DNA. GFP-MTase alone (line), GFP-MTase – 21TH21B DNA complex (dashed line), GFP-MTase – 21T21BH DNA (dotted line). Excitation was at 395 nm. (B) Fluorescence emission scans of 500 nM GFP-MTase showing FRET to 500 nM Dylight549-labelled mutant ocr proteins. GFP-MTase alone (line), GFP-MTase – E20C ocr complex (dashed line), GFP-MTase – S68C ocr complex (dotted line), GFP-MTase – E117C ocr complex (small crosses). Excitation was at 395 nm.

Fig. 2A shows the induction of FRET when the GFP-MTase was bound to a HEX-labelled 21 bp duplex DNA containing the specificity sequence. Note that significantly more energy transfer occurred to the HEX label in duplex 21TH21B (50.1% transfer) than to HEX in the 21T21BH duplex (8.5% transfer) indicating that one end of the duplex was further from the GFP than the other end. Using the calculated Förster distance, the GFP is separated from the HEX label of 21TH21B by 6.10 nm and from the HEX label on 21T21BH by 9.12 nm.

Fig. 2B shows the induction of FRET when the GFP-MTase bound to the mutant ocr proteins labelled with Dylight549. The amount of energy transfer depended on the mutant used. However, since the introduction of a single cysteine into each ocr subunit means that there are two FRET acceptors and, given the elongated shape of ocr, these acceptors are highly likely to be located at different distances from the GFP donor. Hence the observed FRET was a complex average of the two distances given the 1/*r*^6^ dependence of FRET on distance. In the absence of further information, we simply calculated this “average” distance to be 7.62, 10.21 and 6.60 nm for the E20C, S68C and E117E mutant ocr proteins assuming *κ*^2^ is 2/3.

### 3.7 FRET measurements of GFP to HEX using time-resolved fluorescence

The fluorescence decay of the GFP for 1:1 mixtures of DNA and GFP-MTase and of labelled ocr with GFP-MTase were determined. The emission was collected at the magic angle to remove undesirable anisotropy effects on the fluorescence decay and the fitted lifetimes are shown in Table 2.

It was apparent that the presence of the HEX label on DNA or the Dylight549 label on ocr reduced the average fluorescence lifetime, <*τ*>, of the GFP donor. This was indicative of energy transfer and an average distance between the donor and acceptor could be calculated (Table 3). This distance was in all cases except those using the S68C ocr mutant protein, very similar to the distance calculated from the fluorescence intensity data.

It was also clear that the bi-exponential decay of GFP became a three exponential decay in some complexes so changes in individual lifetimes due to FRET could also be calculated. Considering first the bi-exponential decays, we assumed that since the pre-exponential factors remained roughly constant in the presence or absence of acceptor, that FRET shortens the 2.20 ns lifetime to 1.61 or 1.37 and the 3.01 ns lifetime to 2.84 or 2.67 ns for the 21TH21B and S68C samples, respectively, allowing FRET efficiencies and distances to be calculated (Table 3). In the three exponential decays, we assumed that the 3.01 ns lifetime split into two components; the 2.7–2.8 ns component and the ∼0.3 ns component as the sum of the two pre-exponential factors approximately equalled the initial 0.64 pre-exponential factor. The 2.20 ns lifetime, which once again hardly changed its pre-exponential factor, was assumed to decrease to the 1.3–1.4 ns lifetime. These assumptions allowed distances to be calculated. These interpretations imply multiple locations for the GFP with the electronic transition responsible for the 3.01 ns lifetime being particularly sensitive to an interaction with the acceptors. However, the photophysics of GFP and its derivatives is so complex in FRET experiments [22] that it may be wise not to over interpret the distances calculated from the individual lifetimes, particularly since there are two acceptors on the ocr mutant proteins, but rather to use the distance from the average lifetime when examining the location of GFP on the MTase structural model. This is particularly the case for the FRET between GFP and the label in the S68C ocr mutant protein where the acceptor was not free to rotate.

## 4 Discussion

Our results show that it is possible to fuse GFP to the C-terminus of HsdS in a Type I MTase without any deleterious effect on *in vivo* methylation or *in vitro* binding to either DNA or to a DNA mimic. The assembly of the trimeric MTase is also not affected because the GFP appears to be able to adopt a range of conformations with respect to the MTase and freely move between them.

**Fig. 3:**
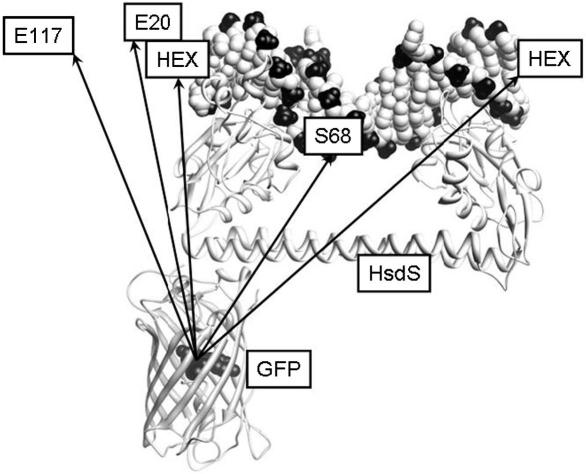
The HsdS subunit bound to a DNA duplex as proposed from electron microscopy data [7] is shown above a GFP model with the chromophore shown in the centre of the GFP β-barrel. The locations of the HEX labels (21TH21B is on the left and 21T21BH is on the right) and of the locations of the ocr residues labelled with Dylight549 are indicated (ocr is not shown but superimposes on and extends further out than the DNA duplex shown). The arrows show the FRET distances determined from <*τ*> given in Table 3 except for the distance to S68 on ocr where the distance in the actual model is shown (the FRET distance is longer for this pair but is incorrect due to rotational constraints on the acceptor).

Recently Kennaway et al. [7] have published an atomic model of the M.EcoKI MTase bound to a DNA duplex and to ocr. Fig. 3 shows the HsdS subunit bound to DNA with the GFP chromophore placed roughly at the distances determined by FRET using the average fluorescence lifetimes (the ocr protein roughly takes the place of the DNA in the atomic model of M.EcoKI and ocr). It can be seen that the results all converge on approximately the same location for the GFP apart from the distance to the S68C location on the ocr protein. The GFP is best located directly below one end of the HsdS subunit to satisfy the FRET distances. This location is what would be expected from the model of M.EcoKI MTase as the location of the C-terminus of HsdS.

Given the similarity in the structures of the Type I R/M enzymes, this GFP-fusion strategy should work for the other well studied Type I R/M enzymes such as EcoR124I and EcoAI and will facilitate single-molecule experiments both *in vitro* and *in vivo*. It will also allow fluorescence microscopy of the R/M systems in living cells and we note that overexpression of the fusion protein turns the cytoplasm of *E. coli* bright green (unpublished results).

## Figures and Tables

**Table 1:** Modification of phage *λ*_v.o_ by GFP-MTase protects the phage DNA against the EcoKI R/M system.

Phage recovered from NM1261(DE3) containing the following plasmids	Strain used for plating of recovered phage	Titre of phage on plating strain (pfu/ml)
pBIO2	NM1261(DE3) r^−^m^−^	3.0 × 10^8^
pBIO2	NM1049(DE3) r^+^m^+^	1.4 × 10^4^
pJFMS	NM1261(DE3) r^−^m^−^	0.8 × 10^8^
pJFMS	NM1049(DE3) r^+^m^+^	1.2 × 10^8^
pJFMSEGFP	NM1261(DE3) r^−^m^−^	1.4 × 10^8^
pJFMSEGFP	NM1049(DE3) r^+^m^+^	1.5 × 10^8^

**Table 2:** Time-resolved fluorescence decay analysis of samples showing FRET between GFP and HEX or Dylight549. Excitation at 405 nm, emission at 510 nm. The pre-exponential factor for each lifetime is given in the brackets.

Sample	*τ*1(ns)	*τ*2(ns)	*τ*3(ns)	*χ*^2^	<*τ*>(ns)
GFP-MTase		2.20 (0.36)	3.01 (0.64)	1.082	2.72
GFP-MTase + 21TH21B DNA	0.29 (0.38)	1.42 (0.29)	2.84 (0.33)	1.076	1.45
GFP-MTase + 21T21BH DNA		1.61 (0.31)	2.84 (0.69)	1.061	2.46
GFP-MTase + E20C ocr	0.28 (0.27)	1.42 (0.40)	2.72 (0.33)	1.052	1.55
GFP-MTase + S68C ocr		1.37 (0.44)	2.67 (0.56)	1.173	2.09
GFP-MTase + E117C ocr	0.33 (0.38)	1.28 (0.32)	2.84 (0.29)	1.012	1.38

**Table 3:** FRET distances (nm) calculated using fluorescence decay times of GFP-MTase in the absence or presence of the fluorescence acceptor compared to distances calculated from fluorescence intensity measurements. All distances are in nm.

Sample	Distance from *τ*3 to *τ*1	Distance from *τ*2 to *τ*2	Distance from *τ*3 to *τ*3	Distance from <*τ*>	Distance from intensity
GFP-MTase + 21TH21B DNA	4.23	6.79	9.80	6.27	6.10
GFP-MTase + 21T21BH DNA		7.26	9.90	8.97	9.38
GFP-MTase + E20C ocr	4.47	7.22	9.48	6.84	7.62
GFP-MTase + S68C ocr		7.10	9.21	7.98	10.21
GFP-MTase + E117C ocr	4.61	6.90	10.4	6.56	6.60

## Appendix A Supplementary data

Supplementary data 1 A document containing figures and tables.
