# Supplementary material for: Fusion of GFP to the M.EcoKI DNA methyltransferase produces a new probe of Type I DNA restriction and modification enzymes
Source: Biochem Biophys Res Commun. 2010 Jul 23;398(2):254–9. doi: 10.1016/j.bbrc.2010.06.069 (PMC2914225; doi:10.1016/j.bbrc.2010.06.069)
Supplement: Supplementary data 1 — A document containing figures and tables. [file mmc1.doc]

**Supplementary information**

**Fusion of GFP to the M.EcoKI DNA methyltransferase produces a new probe of Type I DNA restriction and modification enzymes.**

Kai Chen, Gareth A. Roberts, Laurie P. Cooper, John H. White, David T.F. Dryden ^*^

School of Chemistry, University of Edinburgh, The King’s Buildings, Edinburgh, EH9 3JJ, UK

* Author for correspondence: david.dryden@ed.ac.uk, Tel +44 (0)131 650 4753, FAX +44 (0)131 650 6453

*Plasmid pJFMSEGFP for production of GFP-MTase.*

The expression construct is derived from pJFMS [18] and pEGFP-N1 (Clonetech). The HsdS open reading frame was fused to EGFP by use of the polymerase chain reaction (PCR). PCR using oligonucleotides hsdS1 (5’CATCACTTTCTGCTGGTGC3’) and hsdSEGFPbs (5’CAGCTCCTCGCCCTTGCTCACCATGGATTTTTTACGTGAGGCTTTTTTACCCCC3’) with pJFMS as template, generated a 1kb fragment comprising the 3’ end of *hsdS* fused to the first 24 bases of EGFP. PCR with the second pair of oligonucleotides, hsdSEGFPts (5’GGGGGTAAAAAAGCCTCACGTAAAAAATCCATGGTGAGCAAGGGCGAGGAGCTG3’ and EGFPcterm

(5’GAACTTGAATTCTTAATGATGATGATGATGATGCTTGTACAGCTCGTCCATGCC3’), with pEGFP-N1 as template, fused 30 bases at the 3’ end of *hsdS* to EGFP. The EGFP gene includes a C-terminal polyhistidine tag and EcoRI site.

These two PCR products were purified and fused in a reaction primed with oligonucleotides hsdS1 and EGFPcterm. The resulting product was purified and digested with HindIII and EcoRI. pJFMS was digested with HindIII and EcoRI, purified and then ligated with the PCR product. Recombinant plasmids were isolated from transformed DH5α cells and the desired DNA sequence was confirmed. Finally, the HindIII fragment of pJFMS, containing the entire *hsdM* and the remainder of *hsdS*, was ligated into the HindIII interval of the recombinant plasmid. Orientation of the insert and continuity of the *hsdS* reading frame were confirmed by sequencing. This plasmid was named pJFMSEGFP and we call the protein GFP-MTase.

*Purification of GFP-MTase*

The construct pJFMSEGFP was used to transform *E. coli* BL21(DE3) and the cells were plated on LB agar containing 100 μg/ml carbenicillin. A single colony was picked from the plate to generate a starter culture. The culture was grown at 37ºC to an OD_600_ of ~0.6 before addition of IPTG (final concentration of 1 mM). Growth was then continued for a further 3 h at 37ºC before harvesting the cells by centrifugation (5000 *g*, 15 min, 4ºC). The cell pellet was stored at -20ºC until required. Approximately 15 g of cell pellet was defrosted on ice for 15 mins and resuspended in 50 ml of buffer A (20mM sodium phosphate, 500 mM NaCl, 20mM imidazole, pH 7.4). A protease inhibitor tablet was added to the buffer (*Complete* EDTA-free protease inhibitor, Roche). The cells were disrupted by sonication on ice using a Soniprep 150 sonicator (Sanyo) fitted with a 9mm diameter probe for approximately 15 mins with intermittent cooling between bursts. The extract was centrifuged at 20,000 g for 1 hour at 4^o^C, and the supernatant filtered through a filter unit (0.45μm; Sartorius AG). The clarified extract was then applied to a HisTrap FF 5 ml column (GE Healthcare) equilibrated in buffer A at 3 ml/min. Once the sample was loaded, the column was washed with 100 mls of buffer A. Bound protein was eluted with buffer B (20mM sodium phosphate, 500 mM NaCl, 500 mM imidazole, pH 7.4). Protein elution was monitored by observing the green colour and was completed in 5 mls total volume. Analysis on SDS-PAGE showed there were still impurities present and further purification was necessary. The sample was loaded onto a HiLoad 16/60 Superdex 200 gel filtration column (GE Healthcare) pre-equilibrated in buffer C (20 mM Tris-HCl pH 8.0, 200 mM NaCl, 10 mM MgCl_2_, 7 mM 2-mercaptoethanol) and the protein eluted at 12 mls/hour. The fractions were analysed by SDS-PAGE and those containing the protein were pooled. The protein still contained impurities and was further purified using anion exchange chromatography. The sample was dialysed against 2 litres of buffer D (20 mM Tris-HCl pH 8.0, 10 mM MgCl_2_, 7 mM 2-mercaptoethanol) for 3 hours and then loaded onto a DEAE anion exchange column (30cm x 1.6 cm diameter), equilibrated in buffer D, at a flow rate of 50 ml/h. The column was washed with one column volume of buffer D and the bound protein eluted using a 500 ml gradient of 0 to 0.4 M NaCl in buffer D at a flow rate of 25 ml/h. The green eluted fractions were analysed by SDS-PAGE. The protein had separated out into GFP-M_2_S_1_ and GFP-M_1_S_1_ components as observed for the wild type protein [18], figure S1.The purest fractions for each were pooled separately and concentrated using spin concentrators with a 30 kDa cutoff membrane (VivaScience AG). The purified proteins were stored at -20 ^o^C in solution containing 50% (v/v) glycerol. The final yield was approximately 40 mgs of pure GFP-M2S_1_ and 12 mgs pure GFP-M_1_S_1_.

The extinction coefficient of M.EcoKI at 280 nm is calculated to be 143140 M^-1^ cm^-1^, GFP-MTase at 280 nm is 167560 M^-1^ cm^-1^ and GFP at 396 nm is 27000 M^-1^ cm^-1^. UV-Vis measurements were performed on Varian Cary 50 Bio spectrophotometer.

Figure S1. Purification of GFP-MTase. a. lane 1 shows markers, lane 2 shows eluate from HisTrap column, lanes 2-4 show predominantly GFP-M_1_S_1_ eluting from gel filtration column, lanes 5-7 show predominantly GFP-M_2_S_1_ eluting from the gel filtration column, lane 8 shows pure M_2_S_1_ with HsdM above the HsdS band. In lanes 2-7, the major upper band is GFP-HsdS and the major band below that is HsdM. b. lane 1 shows markers, lanes 2 and 3 show GFP-M_1_S_1_ eluting from the ion exchange column, lanes 4 and 5 show the pure GFP-M_2_S_1_ eluting from the column, lane 6 shows pure M_2_S_1_.

a. b.


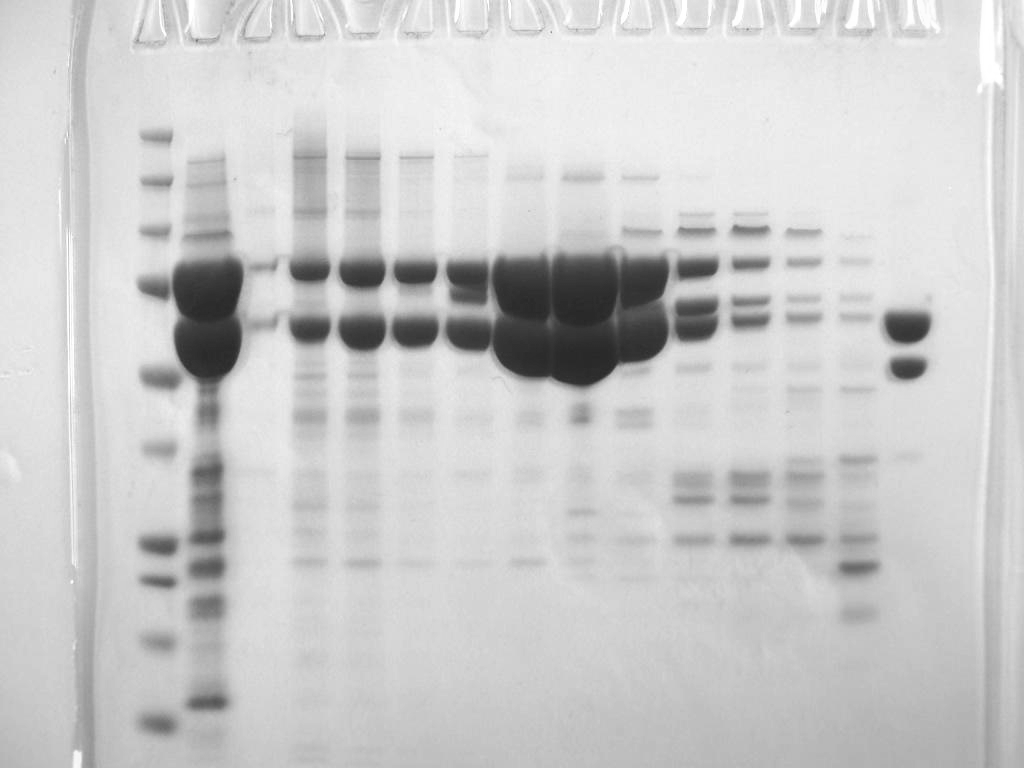


1 2 3 4 5 6 7 8


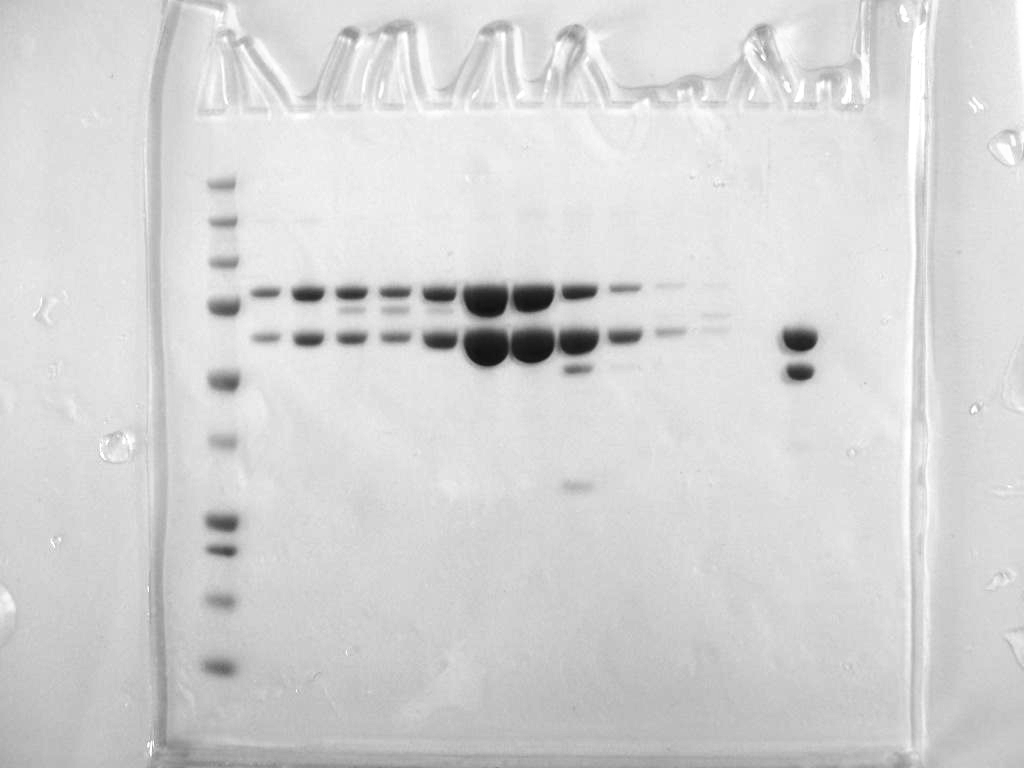


1 2 3 4 5 6

*DNA and ocr binding by GFP-MTase.*

The interaction between GFP-MTase and both ligands was measured to see if it was the same as for the normal MTase.

A continuous variation titration keeping the total concentration of [21TH21B]+[protein] constant at 200 nM showed a maximum amount of FRET at a 1:1 ratio of DNA to GFP-MTase indicative of a 1:1 complex, figure S2a. The binding affinity in the absence of NaCl was 19 +/- 7 nM virtually identical to the 17.7 +/-2.2 nM found for this duplex binding to normal MTase using fluorescence anisotropy [15]. The amount of complex formed decreased as the amount of NaCl increased (data not shown) as observed previously [15].

Normal ocr binds extremely strongly to the MTase and prevents the MTase from dissociating in to its component subunits at concentrations below ~200nM [14]. The complex is stable when passed through a gel filtration column. Passing a 1:1 ratio mixture of labelled E117C-ocr and GFP-MTase through a gel filtration column showed that the major peak of labelled ocr bound to the GFP-MTase and eluted as a complex between 5.5 and 6 minutes, figure S2b. Minor labelled contaminating species eluting around 6.5 minutes did not bind to the GFP-MTase. The other labelled mutant ocr proteins behaved similarly (data not shown). Hence a 1:1 ratio of labelled ocr dimer to GFP-MTase is appropriate.

Figure S2. Interaction stoichiometry between GFP-MTase and HEX-labelled DNA or the labelled ocr mutant proteins. a. Continuous variation titration of the amount of FRET between GFP-MTase and 21TH21B DNA duplex showing maximum FRET at a 1:1 ratio. Excitation was at 395 nm and emission at 520 nm. b. Size exclusion chromatography. Left panel: Dylight549-labelled E117C ocr mutant protein (+ GFP-MTase in dashed line), excitation 550 nm, emission 570 nm. Right panel: Dylight549-labelled E117C ocr mutant protein (+ GFP-MTase in dashed line), excitation 295 nm, emission 350 nm. Protein concentrations were 0.5 μM and the elution data from 5 to 7.5 minutes are shown.

a.

b.

Table S1. Time-resolved fluorescence and anisotropy decays for all fluorophores. The fluorophores were all excited directly rather than via FRET. Figures in brackets are the pre-exponential factors for each lifetime component.

| Sample | τ1 (ns) | τ2 (ns) | τ3 (ns) | χ^2^ | φ (ns) | r_o_ | r__ | χ^2^ | λ_ex_ (nm) | λ_em_ (nm) |
| --- | --- | --- | --- | --- | --- | --- | --- | --- | --- | --- |
| GFP | 3.25 +/- 0.08 |  |  | 1.098 | 14.9 +/- 2.2 | 0.36 +/- 0.03 | 0.13 +/- 0.03 | 1.146 | 405 | 510 |
| GFP-MTase |  | 2.20 +/-0.04  (0.36) | 3.01 +/-0.06  (0.64) | 1.082 | 14.1 +/- 2.5 | 0.31 +/- 0.03 | 0.21 +/- 0.02 | 1.219 | 405 | 510 |
| 21TH21B + 0 mM [NaCl] | 2.62 +/- 0.06 (0.554) | 4.15 +/- 0.06 (0.446) |  | 1.207 | 0.67 +/- 0.26 | 0.134 +/- 0.028 | -0.025 +/- 0.004 | 1.204 | 500 | 565 |
| 21TH21B + 0 mM [NaCl]  + GFP-MTase | 2.91 +/- 0.03 (0.615) | 4.15 +/- 0.04 (0.384) |  | 1.327 | 0.43 +/- 0.11 | 0.210 +/- 0.003 | 0.109 +/- 0.015 | 0.912 | 500 | 565 |
| Ocr E20C-dylight549 | 0.86 +/- 0.01 (0.829) | 1.96 +/-0.05 (0.171) |  | 1.093 | 0.57 +/- 0.04 | 0.166 +/- 0.002 | 0.017 +/- 0.005 | 1.167 | 500 | 570 |
| Ocr E20C-dylight549 + GFP-MTase | 1.30 +/-0.04 (0.553) | 2.47 +/-0.04 (0.447) |  | 0.905 | 0.92 +/- 0.05 | 0.207 +/- 0.020 | 0.160 +/- 0.040 | 1.142 | 500 | 570 |
| Ocr S68C-dylight549 | 0.75 +/-0.01 (0.885) | 2.14 +/-0.04 (0.115) |  | 1.030 | ? | ? | ? | ? | 500 | 570 |
| Ocr S68C-dylight549 + GFP-MTase | 1.21 +/-0.04 (0.548) | 2.56 +/-0.03 (0.452) |  | 0.962 | 12.9 +/- 7.6 | 0.184 +/- 0.004 | 0.103 +/- 0.005 | 0.999 | 500 | 570 |
| Ocr E117C-dylight549 | 0.66 +/-0.01 (0.937) | 1.70 +/-0.06 (0.063) |  | 0.824 | 0.82 +/-0.01 | 0.202 +/- 0.003 | 0.072 +/- 0.004 | 0.949 | 500 | 570 |
| Ocr E117C-dylight549 + GFP-MTase | 0.84 +/-0.01 (0.851) | 2.11 +/-0.04 (0.149) |  | 0.726 | 0.45 +/-0.03 | 0.247 +/- 0.004 | 0.170 +/- 0.008 | 1.178 | 500 | 570 |

*Fluorescence and anisotropy decay of the labelled ocr mutant proteins.*

Figure S3. Anisotropy decays of labelled mutant ocr proteins or HEX-21TH21B DNA in the absence (black) or presence of GFP-MTase (red). Excitation was at 500 nm and emission at 570 nm. The total fluorescence intensity decay curve for the ocr or DNA alone is shown in blue. The anisotropy decays for the S68C ocr are either unusual showing a rise and decay (S68C ocr by itself) or very long (with GFP-MTase) indicating constrained motion of the label. The anisotropy curves for the other labelled mutant ocr proteins and for the HEX-21TH21B DNA show a rapid decay to an approximately constant value. The presence of the GFP-MTase increases the magnitude of this final value due to the increase in mass of the rotating complex.
